# Non contiguous-finished genome sequence of *Pseudomonas syringae* pathovar *syringae* strain B64 isolated from wheat

**DOI:** 10.4056/sigs.3997732

**Published:** 2013-07-30

**Authors:** Alexey Dudnik, Robert Dudler

**Affiliations:** Institute of Plant Biology, University of Zurich, Zurich, Switzerland

**Keywords:** *Pseudomonas syringae*, genome, syringolin, wheat, plant-pathogen interactions

## Abstract

The Gram-negative gammaproteobacterium *Pseudomonas syringae* is one of the most wide-spread plant pathogens and has been repeatedly reported to cause significant damage to crop plantations. Research on this pathogen is very intensive, but most of it is done on isolates that are pathogenic to *Arabidopsis*, tomato, and bean. Here, we announce a high-quality draft genome sequence of *Pseudomonas syringae* pv. *syringae* B64 which is the first published genome of a *P. syringae* strain isolated from wheat up to date. The genome sequence will assist in gaining insights into basic virulence mechanisms of this pathogen which has a relatively small complement of type III effectors.

## Introduction

*Pseudomonas syringae* strains have been isolated from more than 180 host species [1] across the entire plant kingdom, including many agriculturally important crops, such as bean, tomato, cucumber, as well as kiwi, stone fruit, and olive trees. Strains are divided into more than 50 pathovars primarily based on host-specificity, disease symptoms, and biochemical profiles [2-4]. The first strain of this species was isolated from a lilac tree (*Syringa vulgaris*), which gave origin to its name [5].The observed wide host range is reflected in a relatively large genetic heterogeneity among different pathovars. This is most pronounced in the complement of virulence factors, which is also assumed to be the key factor defining host specificity [6]. For successful survival and reproduction, both epiphytic and endophytic *P. syringae* strains deploy different sets of type III and type VI secretion system effectors, phytotoxins, EPS, and other types of secreted molecules [6-11]. Currently, there are three completely sequenced *P. syringae* genomes published: pathovar *syringae* strain B728a which causes brown spot disease of bean [12], pathovar *tomato* strain DC3000 which is pathogenic to tomato and *Arabidopsis* [13], and pathovar *phaseolicola* strain 1448A, causal agent of halo blight on bean [14]. There are also a number of incomplete genomes of various qualities available for other strains.

*Pseudomonas syringae* pv. *syringae* strain B64 was isolated from hexaploid wheat (*Triticum aestivum*) in Minnesota, USA [15]. The strain has been deployed in several studies mainly addressing phylogenetic diversity of *P. syringae* varieties [15-18], but never as an infection model for wheat. The genome sequencing of the B64 strain and its comparison with the other published genomes should reveal wheat-specific adaptations and give insights in virulence strategies for colonizing monocot plants.

## Classification and features

**Figure 1:**
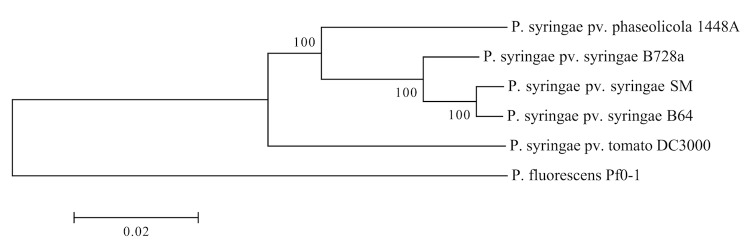
Phylogenetic tree constructed using neighbor-joining method using MLST approach [40] and MEGA 5.10 software suit [41] with 1,000 bootstraps. The tree features the three completely sequenced *P. syringae* model strains *Pto* DC3000, *Pss* B728a, and *Pph* 1448A, the strain *Pss* B64 itself, as well as another wheat-isolated strain *Pss* SM. The model strains represent the major phylogenetic clades of *P. syringae*: I, II and III respectively. *P. fluorescens* Pf0-1 was used as an outgroup. The analysis confirms placement of *Pss* B64 into clade II.

*Pseudomonas syringae* belongs to class *Gammaproteobacteria*. Detailed classification of this species is still under heavy debate. Young and colleagues have proposed to group all plant-pathogenic oxidase-negative and fluorescent *Pseudomonas* strains into a single species, *P. syringae*, which is to be further sub-divided into pathovars [4,19]. Several DNA hybridization studies have shown a large genetic heterogeneity among the groups, however biochemical characteristics, with a few exceptions, did not allow elevating those into distinct species [20,21]. Currently, the species is divided into five phylogenetic clades based on MLST analysis. *P. syringae* pv. *syringae* (*Pss*) strains belong to group II within this nomenclature [22]. The basic characteristics of *Pss* B64 are summarized in Table 1, while its phylogenetic position is depicted in Figure 1.

**Table 1:** Classification and the general features of *P. syringae* pv. *syringae* B64 according to the MIGS recommendations [23]

**MIGS ID**	**Property**	**Term**	**Evidence code^a^**
		Domain *Bacteria*	TAS [24]
		Phylum *Proteobacteria*	TAS [25]
		Class *Gammaproteobacteria*	TAS [26,27]
	Current classification	Order *Pseudomonadales*	TAS [28,29]
		Family *Pseudomonadaceae*	TAS [30,31]
		Genus *Pseudomonas*	TAS [30-34]
		Species *Pseudomonas syringae*	TAS [30,35]
		Pathovar *syringae*	TAS [36]
		Strain B64	TAS [15,16]
	Gram stain	Negative	TAS [37]
	Cell shape	Rod-shaped	TAS [37]
	Motility	Motile	TAS [37]
	Sporulation	None	TAS [37]
	Temperature range	Mesophilic	TAS [38]
	Optimum temperature	28°C	TAS [38]
MIGS-22	Oxygen	Aerobic	TAS [37]
	Carbon source	Heterotrophic	TAS [36]
	Energy metabolism	Chemoorganotrophic	TAS [37]
MIGS-6	Habitat	Host-associated	TAS [17,36,37]
MIGS-6.3	Salinity	Not reported	
MIGS-10	Extrachromosomal elements	None	IDA
MIGS-11	Estimated Size	5.93 Mb	IDA
MIGS-15	Biotic relationship	Parasitic	NAS
MIGS-14	Pathogenicity	Pathogenic	TAS [19]
	Host	*Triticum aestivum*	TAS [15,17]
	Host taxa ID	4565	
	Cell arrangement	Single	TAS
	Biosafety level	1	NAS
	Isolation source	Leaf	NAS
MIGS-4	Geographic location	Minnesota, USA	TAS [15]
MIGS-5	Sample collection time	Not reported	
MIGS-4.1	Latitude	Not reported	
MIGS-4.2	Longitude	Not reported	
MIGS-4.3	Depth	Not reported	
MIGS-4.4	Altitude	Not reported	

^a^Evidence codes - IDA: Inferred from Direct Assay (first time in publication); TAS: Traceable Author Statement (a direct report exists in the literature); NAS: Non-traceable Author Statement (not directly observed for the living, isolated sample, but based on a generally accepted property of the species, or anecdotal evidence). These evidence codes are from the Gene Ontology project [39].

*Pss* B64 has similar physiological properties as other representatives of its genus. It can grow in complex media such as LB [42] or King’s B [43], as well as in various defined minimal media: HSC [44], MG-agar [45], PMS [46], AB-agar [47], and SRM_AF_ [48]. Even though the optimal growth temperature is 28°C, the bacterium can also replicate at 4°C. Growth is completely inhibited above 35°C. *Pss* B64 is capable of endophytic growth in the wheat leaf mesophyll, but does not seem to cause any symptoms unless a very high inoculation dose is applied.

The bacterium has a weak resistance to ampicillin (25 mg/L) and chloramphenicol (10 mg/L). It is also possible to develop spontaneous rifampicin-resistant mutants. In addition, the genomic sequence predicts this strain to be polymyxin B insensitive due to presence of the *arn* gene cluster.

## Genome sequencing information

### Genome project history

The organism was selected for sequencing because it has been identified to have a syringolin biosynthesis gene cluster [49]. Syringolin is a proteasome inhibitor produced by some strains of pathovar *syringae*. As a consequence of proteasome inactivation a number of plant intracellular pathways are being inhibited, including the entire salicylic acid-dependent defense pathway, thus promoting the entry of bacteria into leaf tissue and subsequent endophytic growth [9]. Since up to now it has not been possible to establish an infection model for syringolin in the model plant *Arabidopsis*, it was decided to explore another common research target and one of the most important crop plants, bread wheat (*Triticum aestivum*). The genome project has been deposited in the Genbank Database (ID 180994) and the genome sequence is available under accession number ANZF00000000. The version described in this paper is the first version, ANZF01000000. The details of the project are shown in Table 2.

**Table 2:** Genome sequencing project information

**MIGS ID**	**Property**	**Term**
MIGS-31	Finishing quality	High-quality draft
MIGS-28	Libraries used	3kb paired-end library
MIGS-29	Sequencing platform	Roche Genome Sequencer FLX+
MIGS-31.2	Sequencing coverage	31.8×
MIGS-30	Assembler	Newbler 2.5.3
MIGS-31.3	Contigs	41
MIGS-32	Gene calling method	RAST server
	NCBI project ID	180994
	NCBI accession number	ANZF00000000
	Date of release	January 18, 2013
	GOLD ID	Gc02493
	Database: IMG/ER	2523533564
	Project relevance	Plant-pathogen interactions, model for syringolin effects

### Growth conditions and DNA isolation

*P. syringae* pv. *syringae* strain B64 was grown in 40 mL of LB medium at 28°C, 220 rpm until OD_600_ of ~1.0. Genomic DNA was isolated from the pelleted cell using a Qiagen Genomic-tip 100/G column (Qiagen, Hilden, Germany) according to the manufacturer’s instructions.

### Genome sequencing and assembly

A 3kb paired-end library was generated and sequenced at the Functional Genomics Center Zurich on a Roche Genome Sequencer FLX+ platform. A total of 872,570 high-quality filtered reads with a total of 188,465,376 bases were obtained, resulting in 31.8-fold average sequencing coverage. The obtained reads were assembled *de novo* using Newbler 2.5.3. This resulted in 150 contigs combined into one 6 Mb-long super-scaffold and 3 smaller scaffolds of 5.29 kb, 2.84 kb and 2.74 kb in size. The largest of the minor scaffolds constituted a ribosomal RNA operon, the other two showed sequence similarity to non-ribosomal peptide synthase modules. A portion of intra-scaffold gaps have been closed by sequencing of PCR products using Sanger technology, decreasing the total number of contigs to 41 with a contig N50 value of 329.4 kb, the longest contig being 766.5 kb long. Note that the Genbank record contains 42 contigs due to fact that one of the contigs was split into two parts in order to start the assembly with the *dnaA* gene. While closing gaps it became possible to allocate the positions of all ribosomal operons by sequence overlap and thus to incorporate the largest of the minor scaffolds. However, it was not possible to precisely map the remaining two minor scaffolds. These must be located within two distinct remaining large gaps, but due to insignificance to the project they have been excluded from the assembly.

### Genome annotation

Initial open-reading frame (ORF), tRNA, and rRNA prediction and functional annotation has been performed using the RAST (Rapid Annotation using Subsystem Technology) server [50]. For the purpose of comparison, the genome has also been annotated using Prokka [51], which utilizes Prodigal [52] for ORF prediction (the RAST server utilizes a modified version of Glimmer [53]). Start codons of all the predicted ORFs were further verified manually, using the position of potential ribosomal binding sites and BLASTP [54] alignments with homologous ORFs from other *P. syringae* strains as a reference. Functional annotations have also been refined for every ORF using BLASTP searches against the non-redundant protein sequence database (nr) and the NCBI Conserved-Domain search engine [55]. Functional category assignment and signal peptide prediction was done using the Integrated Microbial Genomes/Expert reviews (IMG/ER) system [56].

## Genome properties

**Figure 2:**
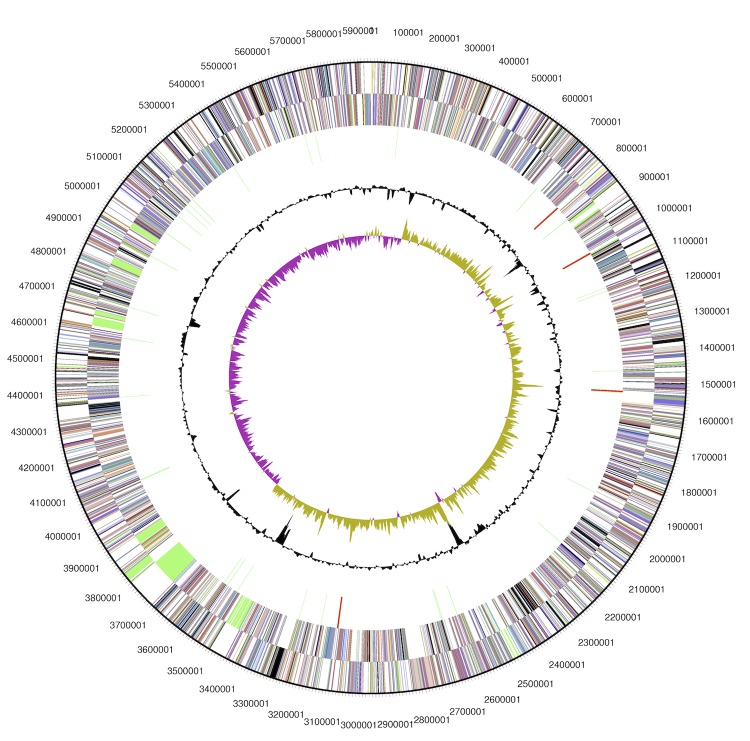
Graphical map of the chromosome. From outside to the center: genes on forward strand (colored by COG categories), genes on reverse strand (colored by COG categories), RNA genes: tRNAs - green, rRNAs - red, other RNAs - black, GC content, and GC skew

The genome of the strain B64 is estimated to be comprised of 5,930,035 base pairs with an average GC-content of 58.55 % (Table 3 and Figure 2), which is similar to what is observed in other *P. syringae* strains [12,13,53]. Of the 5,021 predicted genes, 4,947 were protein coding genes, 4 ribosomal RNA operons, and 61 tRNA genes; 78 were identified to be pseudo-genes. The majority of the protein-coding genes (83.65 %) were assigned a putative function, while the remaining ones were annotated as hypothetical proteins. The distribution of genes into COGs functional categories is presented in Table 4.

**Table 3:** Genome Statistics

**Attribute**	**Value**	**% of Total**
Estimated genome size (bp)	5,930,035	100.00
Estimated total gap length (bp)	56,737	0.96
DNA coding region (bp)	5,146,184	86.78
DNA G+C content (bp)	3,472,195	58.55
Number of replicons	1	-
Extra-chromosomal elements	0	-
Total genes	5,021	100.00
Protein-coding genes^a^	4,869	96.97
RNA genes	74	1.47
rRNA genes	13	0.26
5S rRNA	5	0.10
16S rRNA	4	0.08
23S rRNA	4	0.08
tRNA genes	61	1.21
rRNA operons	4	-
Pseudo-genes	78	1.55
Protein coding genes with function prediction	4,200	83.65
without function prediction^a^	669	13.32
Protein coding genes with COGs	4,013	79.92
with KOGs	1,698	33.82
with Pfam	4,256	84.76
with TIGRfam	1,641	32.68
in paralog clusters	3,933	78.33
Proteins with signal peptides	511	10.18
Proteins with transmembrane helices	1,112	22.15

^a^excluding pseudo-genes; therefore percentage values differ from those displayed at the IMG/ER

**Table 4:** Number of genes associated with the 25 general COG functional categories

**Code**	**Value**	**%age**	**Description**
J	208	4.60	Translation
A	1	0.02	RNA processing and modification
K	352	7.78	Transcription
L	162	3.58	Replication, recombination and repair
B	1	0.02	Chromatin structure and dynamics
D	42	0.93	Cell cycle control, mitosis and meiosis
Y	-	-	Nuclear structure
V	48	1.06	Defense mechanisms
T	332	7.34	Signal transduction mechanisms
M	275	6.08	Cell wall/membrane biogenesis
N	159	3.52	Cell motility
Z	1	0.02	Cytoskeleton
W	-	-	Extracellular structures
U	140	3.10	Intracellular trafficking and secretion
O	156	3.45	Posttranslational modification, protein turnover, chaperones
C	230	5.09	Energy production and conversion
G	265	5.86	Carbohydrate transport and metabolism
E	439	9.71	Amino acid transport and metabolism
F	90	1.99	Nucleotide transport and metabolism
H	177	3.91	Coenzyme transport and metabolism
I	152	3.36	Lipid transport and metabolism
P	268	5.93	Inorganic ion transport and metabolism
Q	122	2.70	Secondary metabolites biosynthesis, transport and catabolism
R	515	11.39	General function prediction only
S	388	8.58	Function unknown
-	1,008	20.08	Not in COGs

The genome contains a complete canonical type III secretion system and ten known effector proteins: AvrE1, HopAA1, HopI1, HopM1, HopAH1, HopAG1, HopAI1, HopAZ1, HopBA1, and HopZ3. Out of these ten, the first five are present in all other sequenced *P. syringae* strains, thereby constituting the effector core, whereas the latter five could be host-determinants for wheat. That there is such a small number of effectors is not something unusual, and is seen in other strains of clade II [22]. In addition, there are two complete type VI secretion system gene clusters and nine putative effector proteins belonging to the VgrG and Hcp1 families. *Pss* B64 genome also encodes gene clusters for biosynthesis of four phytotoxin: syringomycin, syringopeptin, syringolin, and mangotoxin. All of the above-mentioned genome components have been previously demonstrated to be involved in virulence, epiphytic fitness of *P. syringae*, as well as in competition with other microbial species [7-10,57-59]. Additional identified virulence-associated traits are: exopolysaccharides alginate, Psl, and levan biosynthesis, surfactant syringofactin, type VI pili, large surface adhesins, siderophores pyoverdine and achromobactin, proteases and other secreted hydrolytic enzymes, RND-type transporters (including putative *mexAB*, *mexCD*, *mexEF*, and *mexMN* homologs [60,61]), all of which are found in other *P. syringae* strains. It is also notable that *inaZ* gene encoding ice-nucleation protein is truncated by a frameshift, thus making this strain ice-negative. The latter contradicts results of a previous study by Hwang and colleagues [16] in which *Pss* B64 has been identified to be ice-positive. This could be due to an assembly error, or the frameshift could have been introduced at a later point during propagation.
